# Post-inflammatory Pigmentary Alteration (PIPA)-Like Disorder Following COVID-19 Vaccination

**DOI:** 10.7759/cureus.87222

**Published:** 2025-07-03

**Authors:** Larry M Bush, Priscila M Fiallo, Maria T Vazquez-Pertejo

**Affiliations:** 1 Charles E. Schmidt College of Medicine, Florida Atlantic University, Boca Raton, USA; 2 Internal Medicine, Wellington Regional Medical Center, Wellington, USA; 3 Pathology and Laboratory Medicine, Wellington Regional Medical Center, Wellington, USA

**Keywords:** covid-19 infection, cutaneous manifestations, hiv aids, mrna covid-19 vaccination, post-inflammatory pigmentary alteration (pipa), sars-cov-2 infection

## Abstract

SARS-CoV-2 (COVID-19) infection has been associated with various cutaneous manifestations presenting concomitantly with and following clinical resolution of active infection. Similarly, a variety of cutaneous conditions after mRNA-based COVID-19 vaccination, including specific local injection-site reactions, type I hypersensitivity, type IV (delayed) responses, and autoimmune-mediated reactions, has been observed. The commonality between natural infection and vaccination-related skin findings suggests a molecular mimicry of an immunologic nature. We present a case of a previously undescribed incident of cutaneous hyperpigmentation secondary to melanocyte dysfunction and melanin overproduction, triggered by an inflammatory event, in this case, immunization with an mRNA COVID-19 vaccine.

## Introduction

Cutaneous reactions both during and after the resolution of infection caused by SARS-CoV-2 (COVID-19), although not extremely common, are well documented [1,2]. Beginning with the introduction of mRNA COVID-19 vaccines in December 2020 and mass immunization campaigns, there is mounting evidence that these vaccines have the potential to induce a broad spectrum of adverse events limited to the skin [3-5]. The fact that some of these dermatologic manifestations, such as pityriasis rosea-like exanthems, mimic cutaneous conditions associated with COVID-19 infection suggests that the host response to natural infection with the virus is being replicated by the vaccine. Therefore, these manifestations may result from an immune response to the virus rather than a direct viral effect [6,7]. Local injection-site reactions, such as erythema, edema, induration, pruritus, and pain, with onset a few minutes to a few days after COVID-19 vaccination, have been well documented in both clinical trials and real-world clinical practice [6]. In most published case series, patients who developed cutaneous reactions did so more often after having received the first dose, with only a minority experiencing recurrent reactions following the second injection [3,8]. In addition to type I and type IV (COVID arm) hypersensitivity reactions, autoimmune-mediated skin findings, functional angiopathies, and reactivation of viral conditions (e.g., herpesviruses) may be associated with COVID-19 vaccination [9-11]. Many of these reactions are immunological or autoimmunological in nature. We present a case, which, to the best of our knowledge, is a previously undescribed incident of post-inflammatory pigmentary alteration (PIPA)-like disorder. This condition is a cutaneous hyperpigmentation secondary to melanocyte dysfunction with melanin overproduction triggered by an inflammatory event, in this case, immunization with an mRNA COVID-19 vaccine.

## Case presentation

**Figure 1:**
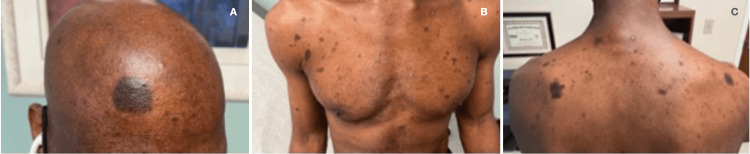
A: Hyperpigmented macular lesions in forehead; B: chest and upper extremities; and C: upper back.

**Figure 2:**
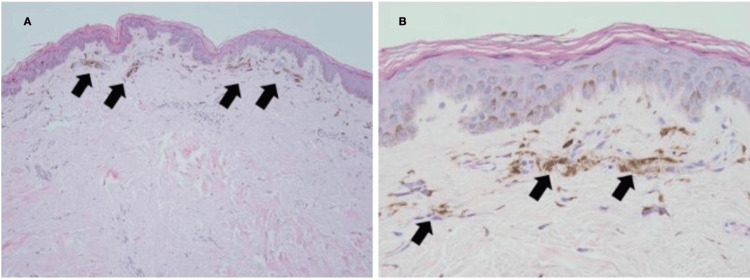
Skin biopsy histopathology. A: Low-power magnification of melanin pigment accumulation in the superficial dermis (arrows; H&E stain, 100x). B: High-power magnification of dermal melanophages (melanin-containing macrophages; H&E stain, 400x).

A 50-year-old man with acquired immunodeficiency syndrome (AIDS) because of infection with an extremely drug-resistant human immunodeficiency virus (HIV) strain (>250,000 RNA copies, CD4- and T-cell lymphocytes <1%, <20/mm^3^) presented with numerous non-pruritic, non-painful, hyperpigmented flat skin lesions of various sizes throughout his body (sparing palms and soles) (Figures 1A, 1B, 1C). He reported the onset of lesions beginning two weeks after the administration of a booster COVID-19 mRNA (mRNA-1273) vaccine (Moderna, Cambridge, MA). He reported no other new clinical signs, symptoms, exposures, or changes in his medical regimen. The remainder of his physical examination was unremarkable. A surgically obtained histologic sample (Figures 2A, 2B) demonstrated aggregates of melanin-laden macrophages (known as melanophages) in the superficial dermis, but without inflammatory infiltrates or dermoepidermal injury. Given the patient’s development of this cutaneous process in close temporal relationship to having received the COVID-19 vaccine, as well as lack of a viable alternative etiologic explanation, a diagnosis of PIPA-like disorder was favored. The absence of histologic inflammatory injury in this patient may be attributed to his AIDS diagnosis and paucity of T-cell lymphocyte response. Immunofluorescence studies to exclude vasculitis, as well as immunohistochemical stains for human herpesvirus 8 (Kaposi’s sarcoma-associated herpesvirus), were negative. Aside from the application of aloe vera skin care lotion, no specific treatment was provided. Over the next four months, no additional skin lesions appeared, and those that were initially present continued to fade, leaving only minimal residual hyperpigmentation. He was advised to refrain from future mRNA COVID-19 vaccinations because of a potentially increased risk of cutaneous injury.

## Discussion

Current literature describes an array of potential post-mRNA COVID-19 vaccine-related cutaneous reactions, as well as systemic inflammatory and immunologic conditions. The list of cutaneous conditions is extensive and includes, but is not limited to, exanthemas, urticaria, eczematous dermatitis, vascular lesions (i.e., leukocytoclastic vasculitis, purpura/petechiae), and autoimmune bullous reactions (i.e., subepidermal bullous disease and pemphigus) [1,2]. In addition to new-onset conditions, the literature also describes post-COVID-19 vaccine flare-ups of preexisting dermatoses such as plaque psoriasis and atopic dermatitis, among many others [4,5]. The time between receiving the vaccine and the onset of reaction ranges from immediate (within minutes) to hours to weeks. Most cutaneous reactions are mild, self-limiting, and resolve shortly after onset. PIPA, also known as “acquired hypermelanosis,” is a hyperpigmentation disorder resulting from either excessive melanin production or atypical melanin deposition in the epidermis or dermis that may occur after cutaneous inflammation or injury [12-14]. The observed higher incidence in darker-skinned individuals (Fitzpatrick skin types III-VI) is attributed to the tendency for melanocytes in skin of color to exhibit labile responses to such conditions or events [15,16]. The pathophysiology can be described by melanin pigment accumulation in response to an inflammatory trigger, resulting in hyperpigmentation through one or a combination of two separate mechanisms [17]. One pathway involves epidermal melanocyte stimulation secondary to an epidermal inflammatory process (e.g., dermatitis) and release of inflammatory mediators (e.g., chemokines, prostanoids), leading to an increase in melanin production and transfer to neighboring keratinocytes [15]. The other, deeper process, referred to as dermal melanosis, involves disruption of the basal cell layer keratinocytes, resulting in abnormal melanin dispersion by leakage from the epidermis to the dermis, where it is then phagocytized by dermal macrophages. PIPA has been associated with various stimuli, including acne vulgaris, cutaneous bacterial and fungal infections, allergic or immunologic reactions (e.g., atopic or contact dermatitis, insect bite reactions), papulosquamous disorders (e.g., lichen planus, psoriasis), and physical or mechanical injury (e.g., radiation, chemical peels) [18]. In our patient, the lack of inflammatory cells in the dermis and/or dermoepidermal junction can be explained by his chronic lymphopenia secondary to AIDS. Nonetheless, melanin pigment was abundant in the dermal macrophages, suggesting an antibody-mediated pathogenesis. Treatment is generally aimed at eliminating the underlying inciting inflammatory entity if still active, along with the use of sunscreen products for photoprotection. Other modalities with various reported rates of success include the application of topical depigmenting agents (e.g., hydroquinone, retinoids, azelaic acid), topical corticosteroids, and cosmetic camouflaging products. Glycolic, salicylic, and trichloroacetic acid peels, as well as laser and light therapies, may also be considered in refractory cases [19,20]. Epidermal hyperpigmentation typically resolves within six to twelve months. However, none of these treatment options have been proven effective for deeper dermal hyperpigmentation, which may be permanent. Morbidity is limited to psychological distress.

## Conclusions

Given this patient’s development of PIPA in close temporal relationship to having received the COVID-19 vaccine (approximately two weeks), the lack of a viable alternative etiologic explanation as to why he developed this cutaneous process, and the current literature describing various post-COVID-19 vaccine reactions, we propose that PIPA can be added to the already extensive list of potential post-mRNA COVID-19 adverse dermatologic events. While most previously documented cutaneous reactions to COVID-19 vaccines are self-limited and of brief duration, our patient’s case adds what was, until now, a previously unrecognized entity, PIPA, to the growing list of potential vaccination-related dermatologic adverse events. The documented chronology of the reaction soon after vaccination, coupled with the histologic results, strongly supports the conclusion that the vaccine induced an immunological response, even amid considerable immunosuppression. Moreover, this case serves as a supplement to the expanding registry of recognized events, both adverse and otherwise, believed to be secondarily associated with the global introduction of mRNA vaccines into clinical immunization practice. Heightened awareness of such atypical presentations may assist in future diagnosis, management, and vaccine-related counseling, particularly in immunocompromised individuals.
